# Effect of a Prebiotic Formulation on Frailty Syndrome: A Randomized, Double-Blind Clinical Trial

**DOI:** 10.3390/ijms17060932

**Published:** 2016-06-14

**Authors:** Cristina Buigues, Julio Fernández-Garrido, Leo Pruimboom, Aldert J. Hoogland, Rut Navarro-Martínez, Mary Martínez-Martínez, Yolanda Verdejo, Mari Carmen Mascarós, Carlos Peris, Omar Cauli

**Affiliations:** 1 Department of Nursing, University of Valencia, Valencia 46010, Spain; cristina.buigues@uv.es (C.B.); julio.fernandez@uv.es (J.F.-G.); rut.navarro@uv.es (R.N.-M.); 2 Natura Foundation, Numansdorp 3281 NC, The Netherlands; cpni.pruimboom@icloud.com; 3 University of Groningen, University Medical Center Groningen (UMCG), Groningen 9712 CP, The Netherlands; 4 Bonusan Besloten Vennootschap, Numansdorp 3280 AA, The Netherlands; a.hoogland@bonusan.nl; 5 GeroResidencias La Saleta, Valencia 46015, Spain; mmartinez@lasaleta.com (M.M.-M.); supelpuig@saleta.com (Y.V.); supcampolivar@saleta.com (M.C.M.); betera@lasaleta.com (C.P.)

**Keywords:** fatigue, biomarker, leucocytes, inflammation, aging

## Abstract

Aging can result in major changes in the composition and metabolic activities of bacterial populations in the gastrointestinal system and result in impaired function of the immune system. We assessed the efficacy of prebiotic Darmocare Pre^®^ (Bonusan Besloten Vennootschap (BV), Numansdorp, The Netherlands) to evaluate whether the regular intake of this product can improve frailty criteria, functional status and response of the immune system in elderly people affected by the frailty syndrome. The study was a placebo-controlled, randomized, double blind design in sixty older participants aged 65 and over. The prebiotic product was composed of a mixture of inulin plus fructooligosaccharides and was compared with placebo (maltodextrin). Participants were randomized to a parallel group intervention of 13 weeks’ duration with a daily intake of Darmocare Pre^®^ or placebo. Either prebiotic or placebo were administered after breakfast (between 9–10 a.m.) dissolved in a glass of water carefully stirred just before drinking. The primary outcome was to study the effect on frailty syndrome. The secondary outcomes were effect on functional and cognitive behavior and sleep quality. Moreover, we evaluated whether prebiotic administration alters blood parameters (haemogram and biochemical analysis). The overall rate of frailty was not significantly modified by Darmocare Pre^®^ administration. Nevertheless, prebiotic administration compared with placebo significantly improved two frailty criteria, e.g., exhaustion and handgrip strength (*p* < 0.01 and *p* < 0.05, respectively). No significant effects were observed in functional and cognitive behavior or sleep quality. The use of novel therapeutic approaches influencing the gut microbiota–muscle–brain axis could be considered for treatment of the frailty syndrome.

## 1. Introduction

Health care for older people in western societies, with high rates of chronic diseases and mental and physical impairment, has become a challenge for every health professional. Frailty is a geriatric syndrome describing physical and functional decline that occurs as a consequence of certain diseases (e.g., cancer, chronic infection, *etc.*) but also even without disease. This syndrome is characterized by an increased risk for poor outcomes related with accidental falls, fractures, disability, comorbidity, health care expenditure and premature mortality [1]. In a recent publication, our group has shown [2] that an increase in neutrophil count and a decrease in lymphocyte count are significantly associated with less hand grip strength and low physical activity, two criteria of frailty syndrome [3]. We wish to test with a confident perspective that any measure that alters the number of leukocytes (decrease in neutrophil count and increase in lymphocyte count) can prevent the progression of frailty syndrome since it has been consistently demonstrated that proper immune function in aging is associated with the absence of other signs and symptoms [4]. The gut microbiota and their metabolites play a central role in modulating gut health and disease [4,5] in all age groups, especially in elderly people [6]. In the elderly, there seems to be a decline in microbiota diversity [7] with lower numbers of bifidobacteria, an increase in Enterobacteriaceae [8] and certain Proteobacteria. Moreover, these changes are suspected to play a role in the causation of bowel disease [9]. Age-related changes in gastro-intestinal physiology and function, such as greater permeability of mucosal membrane, reduced transit times, and secretion of acids by the gastric mucosa, can result in a significant change in the composition of the intestinal microbiota, marked by a decline in bifidobacterial numbers and an increase in putatively detrimental populations such as clostridia and enterobacteria [10,11,12]. This altered composition of the gut microbiota can affect the immune system causing profound and multifaceted changes in the elderly [13]. Prebiotics are food ingredients that, when selectively fermented, may alter the composition and/or activity of the intestinal microbiota and bring possible benefits to the individual’s health. Oral administration of formulas containing prebiotics and fermented milk products may improve intestinal microbiota in this population and may contribute to the regulation of the gastro-intestinal functions and activity of the immune–gut system [14]. The potential use of prebiotics as a modulator of the immune function in older individuals has been suggested [15]. The rotational of our clinical trial was based on previous *in vitro* studies that demonstrated the ability of prebiotics to regulate the immune response through lymphocyte regulation and subsequently the inflammatory response [16,17,18]. Recently, studies performed in healthy humans found that administration of prebiotics increase the percentages of some lymphocyte subtypes [19,20].

Considering that alterations in neutrophil and lymphocyte counts are associated with frailty syndrome and, particularly with two frailty criteria, poor muscular strength and low physical activity [2], the primary goal of our study was to test whether the administration of the prebiotic formulation, Darmocare Pre^®^ (Bonusan Besloten Vennootschap (BV), Numansdorp, The Netherlands), improves frailty syndrome in older individuals by altering the number of neutrophil and lymphocyte counts in the blood. In addition, as secondary outcomes, we evaluated whether Darmocare Pre^®^ administration improves functional capacity, and some aspects usually present in frail older people, e.g., poor quality of sleep, disabilities in daily functioning, and cognitive function.

## 2. Results

### 2.1. Design and Study Population

**Figure 1:**
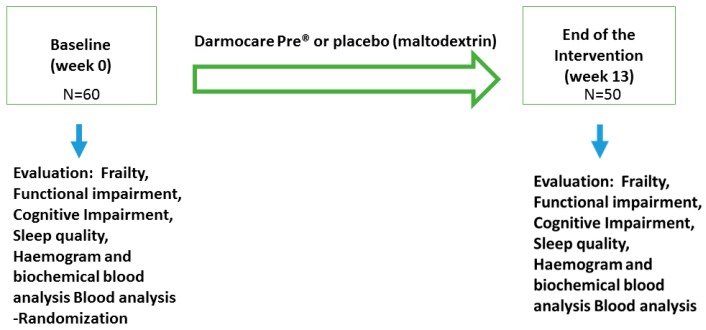
Design of the clinical study. N: number of individuals.

The clinical trial was conducted in 60 volunteers. The participants lived in nursing homes and were non-demented and able to walk (see inclusion and exclusion criteria in the Experimental Section). They were randomized to a parallel group intervention of 13 weeks’ duration with a daily intake of Darmocare Pre^®^ or placebo (Figure 1). Allocation of the participants was blinded. The intervention group used Darmocare Pre^®^. The composition of Darmocare Pre^®^ was Inulin min. 3375 mg fructooligosaccharides (FOS) min. 3488 mg of per level measuring spoon (of 7.5 grams): Excipients: none guaranteed to contain no genetically modified organisms, maize, soy, yeast, gluten, lactose, added saccharose, gelatine, animal substances, preservatives, artificial coloring, flavoring and aromatic substances. The placebo group used an indistinguishable placebo product, which consisted of a corresponding dose of maltodextrin. The mean energy intake in Darmocare Pre^®^-group was 1720 ± 184 kcal/day, and in the placebo group was 1658 ± 220 kcal/day. We did not observe any statistically significant difference (*p* = 0.83) between the two experimental groups regarding the energy intake. Power calculation for the study was estimated as a function of the differences in the measure between the groups that should be detected, and the standard deviation of these values, the *p*-value (α value), and sample size. The probability is 90 percent that the study will detect a treatment difference at a two-sided 0.05 significance level, if the true difference between treatments is 1.000 units. This is based on the assumption that the standard deviation of the difference in the response variables is two. The standard deviation for frailty criteria that we considered for the intervention was two (based on trial aimed to reduce frailty syndrome as a primary outcome). Thus, to achieve 90% power at a two-sided 5% significance level, and to detect a minimum difference of one piece of frailty criteria between placebo and Darmocare Pre^®^-groups, it was calculated that a sample size of a total of 44 patients will enter this two-treatment crossover study [21]. Therefore, to allow for dropouts of people that could not complete the investigation, it was planned to recruit 60 volunteers.

Both prebiotic formulation and placebo were administered after breakfast (between 9–10 a.m) dissolved in a glass of water, which was carefully stirred just before consumption. The primary outcome measured was the effect of treatment on several frailty criteria: weight loss, self-reported exhaustion, low physical activity (PA) level, slowed motor performance and weakness according to Fried *et al.* [3] and as explained below. The secondary outcome was the effect on functional activity assessed by the Barthel Index, cognitive impairment by the Mini-Mental examination score and subjective quality of sleep by the Athens Scale. Moreover, we also analyzed if treatment influenced the values of common blood analytical parameters and the haemogram. Enrollment of participants and evaluations at baseline (week 0, see Figure 1) was performed during the first four months of 2015. Randomization was carried out once the enrollment and baseline evaluations were completed. The intervention took place between June–September in 2015. The participants were selected based on inclusion and exclusion criteria (see study population paragraph). Baseline evaluation was performed 1–2 months before randomization. Within one month after completion of the study, all participants were re-evaluated (frailty syndrome, functional, cognitive and sleep impairment), blood was drawn and faecal samples were collected. The present analyses included the administration of Darmocare Pre^®^ or placebo during 13 week daily administration as described below.

### 2.2. Sample and Dropouts

A total of fifty participants completed the study (83.3%) (15 men and 35 women). The mean age of participants was 73.8 ± 1.6 standard error of the mean (SEM) (range 66–90 years). No significant difference (*p* = 0.74) was observed between the mean age of individuals in the Darmocare Pre^®^ (74.2 ± 1.6) compared to the placebo group (73.4 ± 1.8). No significant difference was found regarding the distribution of sexes in the experimental groups (nine men and 19 women in the Darmocare Pre^®^ and six men and 16 women in the placebo group). Dropout rates were significantly (*p* < 0.05) different between groups (eight participants in the Darmocare Pre^®^ group stopped the treatment and two participants from the placebo group). Four participants from the Darmocare Pre^®^ dropout for cramps, two participants for episodes of diarrhea and two for unknown reasons. One participant that dropped out from the placebo groups suffered from cramps, and the other one stopped for unknown reasons. Among participants that concluded the trial, nine of 22 in the Darmocare Pre^®^ group referred to the appearance of gas, which lasted between 3–8 days. In the placebo group (maltodextrin), none of the participants that concluded the trial referred to any side effects. The two experimental groups (Darmocare Pre^®^ or placebo) were not significantly different at baseline (see Table 1).

### 2.3. Effect of Darmocare Pre^®^ Administration on Frailty Criteria

At baseline and at the end of treatment, participants fulfilled 1–4 frailty criteria according to Fried *et al.* [3]. The overall rate of frailty was not significantly modified by Darmocare Pre^®^ administration. Mean frailty score was 2.8 ± 1.0 at baseline and 2.5 ± 0.8 after intervention in the Darmocare Pre^®^ group and 2.9 ± 0.7 at baseline and 2.8 ± 0.8 after intervention in the placebo group. However, when we analyzed the effect on each of the frailty criteria separately, we observed a significant effect of Darmocare Pre^®^ administration. There was a significant (*p* < 0.05, Mann–Whitney test) improvement (Table 1) in exhaustion and muscle strength in the group of patients who received Darmocare Pre^®^ compared to the placebo group. Exhaustion and muscle strength were not significantly different between groups at baseline (before intervention, see Table 1). The other criteria of frailty syndrome *i.e.*, weight loss, slow walking speed and reduced physical activity were not significantly different between groups neither at baseline nor after the intervention (Table 1). The group of patients who took the prebiotics showed a reduction in fatigue score from 1.4 ± 1.7 at baseline to 0.8 ± 1.4 after intervention (*p* < 0.05) and, in turn, an increase in muscle strength was also observed (measured with hand strength) from 10.6 ± 8.2 at baseline to 12.4 ± 3.2 (*p* < 0.05) after intervention. This strength increase occurred in fact in the dominant hand in 100% of the participants in the Darmocare Pre^®^ group.

We observed an improvement in some other parameters of the frailty syndrome among the group of patients taking prebiotics when comparing values at baseline and after the intervention. We measured an improvement in walking speed in the intervention group (from 8.4 ± 6.0 s at baseline to 7.9 ± 4.5 s. after intervention) *versus* an almost equal result in the placebo group before and after intervention (from 8.6 ± 9.0 at baseline to 8.7 ± 4.2 after). However, this improvement did not reach statistical significance. No significant differences were observed in the Athens scale for subjective sleep quality after Darmocare Pre^®^ administration. The score in Athens scale was 4.18 ± 4.73 at baseline and 4.00 ± 4.38 after intervention in the Darmocare Pre^®^ group; 3.4 ± 3.0 at baseline and 4.5 ± 5.3 after intervention in placebo groups (Table 1). No significant differences were observed in the evaluation of functional activity measured with the Barthel Scale: 74.6 ± 17.7 at baseline and 77.1 ± 29.9 after intervention, in the Darmocare Pre^®^ group, and 76.2 ± 13.0 at baseline and 78.3 ± 13.9 after intervention in the placebo group. No significant differences were found for the mini mental state examination (MMSE), 26.5 ± 3.1 at baseline and 26.4 ± 2.2 after intervention in the Darmocare Pre^®^ group; and 26.1 ± 2.2 at baseline and 25.9 ± 2.1 after intervention in the placebo group.

### 2.4. Effect of Darmocare Pre^®^ on Blood Analytical Parameters

We analyzed several parameters in blood: leukocytes, neutrophils, lymphocytes, monocytes, eosinophils, basophils, platelets, erythrocytes, haemoglobin, glucose, glutamic oxaloacetic transaminase (GOT), glutamic-pyruvate transaminase (GPT), high-density lipoproteins (HDL) cholesterol, low-density lipoproteins (LDL) cholesterol, triglycerides, total proteins, creatinine calcium, sodium and potassium. As shown in Table 2, no significant differences were observed between Darmocare Pre^®^ and placebo group (significance at *p* < 0.05). At baseline, no significant differences were observed in these analytical parameters between the two groups (data not shown).

## 3. Discussion

Improving the function of the immune–gut axis in the elderly has been proposed as an important target for improving health through reduced disease risk and reducing the progression of frailty syndrome that represents the previous steps towards disability, dependence, morbidities and mortality in adults [18]. One of the interventions to improve gut functions are the administration of prebiotics, natural available or synthetic oligosaccharides such as fructooligosaccharides (FOS) and galactooligosaccharides (GOS). Several studies found that administration of prebiotics provide beneficial effects on health [18,22,23]. Based on this finding, we designed a randomized, placebo-controlled study in order to assess the efficacy in frailty syndromes in older individuals, as these individuals often display gastrointestinal problems and frailties that both are characterized by immune dysfunctions among other physiopathological alterations [2,3,4,18,24]. Our results show the following two main results: (1) prebiotic administration as Darmocare Pre^®^, consisting of a mixture of inulin and fructooligosaccharides administered orally for 13 weeks, has a significant and beneficial effect on two criteria of frailty syndrome in older people age 65 and over, namely, hand grip and exhaustion; and (2) prebiotic administration is a safe therapeutic treatment with few and tolerable side effects.

Frailty is considered a syndrome characterized by poor muscle strength that is considered highly prevalent in old age and represents a high risk factor for falls, disability, hospitalization, and mortality [3,24]. In our study, we measured frailty criteria in each participant [3] using muscle weakness, measured by grip strength, as one of the criteria that identify frailty and sarcopenia [25,26,27]. The molecular mechanisms leading to poor muscular strength in frail individuals have not been addressed but inflammation and inappropriate nutritional status likely play a role [28]. Our study results showed a beneficial effect on muscular hand strength after Darmocare Pre^®^ administration induced a beneficial effect on muscular hand strength compared to the placebo group, suggesting that prebiotics may influence the muscular system. Interestingly, a recent multicentric observational study carried out in institutionalized older adults (age > 70) in Spain found that oral nutritional supplement with prebiotic fiber (30% inulin, 70% fructooligosaccharides) for 12 weeks also improved handgrip strength [29]. However, in this latter study, the administration of prebiotics was combined with supplementation of vitamin D and calcium, and thus no conclusion could be made about which factor was responsible for this improvement. The improvement in muscular strength afforded by the prebiotic preparation may also explain the reduced feeling of exhaustion since the two features may likely be closely related. Several mechanisms could account for the improved muscular strength and reduced fatigue found in the subjects treated with the prebiotics. For instance, the reduction in cytokine production [23] might account for these effects [30,31]. Schiffrin *et al.* [32] found, in a prospective, randomized, double-blind, controlled study performed in community dwelling elderly, that oral nutritional supplementation with oligosaccharides (fructooligosaccaride 1.3 g/250 mL) attenuates the expression of mRNA for pro-inflammatory cytokines tumor necrosis factor α (TNF-α) and IL-6 mRNA in blood leucocytes, whereas no significant effects were detected in the fecal gut flora or in the nutritional parameters [32]. Prebiotics may have several effects upon immune function, including the growth of beneficial bacteria leading to inhibition of pathogenic growth, thereby reducing the stimulation of other proinflammatory cytokines (IL-6, IL-1) [22] or reducing activation of macrophages [33,34]. In order to shed some light on the mechanisms by which prebiotic administration exerts its beneficial effects in frailty syndrome, we measured the concentration of C-reactive protein whose levels generally rise in response to acute inflammation, and we did not find any significant differences between groups. We also evaluated the concentration of the pro-inflammatory cytokine TNF-α, but we did not find any significant difference in the concentration of TNF-α between prebiotic and placebo groups. Other cytokines may be involved in fatigue in frail individuals, and future studies should investigate any possible biomarker of this effect. We and other groups have previously published that frailty syndrome is associated with a change in leukocyte counts, and, in particular, in neutrophil and lymphocyte counts [2,35,36], suggesting that interventions that modify neutrophil and/or lymphocyte counts may represent new therapeutic strategies in frailty syndrome. Supporting this goal, it has been demonstrated, at least in healthy humans, that the administration of the prebiotic β2-1 fructan increased the percentages of lymphocyte counts in blood (CD3+CD8+ and CD8+ subtypes) compared to the placebo group [19]. Administration of a formulation containing three prebiotics including galacto-oligosaccharides (also present in Darmocare Pre^®^) for 28 weeks increases the white blood cell counts in blood [20]. For the reasons above, we postulated that prebiotic administration could enhance, among other mechanisms, the number of lymphocytes in the blood of frail older individuals, and, in turn, improve frailty syndrome. However, we were not able to find any significant changes in leukocyte counts in those individuals treated with Darmocare Pre^®^, suggesting that the positive effect obtained by prebiotic administration in frailty was not related to a modulation of leukopoyesis but likely to influence levels of inflammatory mediators or metabolism. Gut microbiota may directly influence muscle cells by several molecular pathways that could potentially mediate the gut microbiota–muscle axis such as energy sparing from the diet, and in the regulation of host immunity and metabolism [23,37]. Thus, this needs to be evaluated in future. Scientific evidence from these reports and our results suggest that prebiotic administration can be an attractive tool to improve health in older individuals, probably through their effects on the modulation of the immune–gut system [27]. The second main result of the clinical trial refers to the safety of Darmocare Pre^®^ administration in frail older individuals, which enables its use with little medical surveillance. Prebiotic administration did not significantly alter cognitive function (assessed by the MMSE), functional impairment (Barthel index), and participants did not refer to any sleep change (Athens scale) during the course of the treatment. However, it should keep in mind that even MMSE is extensively used in clinical practice, including in the primary care setting, as a screening test for dementia [38,39]. Thus, it could not be a sensitive tool to detect small changes in cognition after prebiotic administration. We cannot rule out that other tests more sensitive to detect cognitive alterations in non-demented people would have produced some effects considering the proposed role of immune–gut system of brain functions. During the course of intervention with Darmocare Pre^®^, only modest side effects such as flatulence and cramping occurred in about 30% of individuals, and these symptoms disappeared during treatment, thus suggesting that administration of prebiotics could be well- tolerated and likely usable in those cases of persistence with administration of drugs for the relief of abdominal discomfort and flatulence. In around 4% of cases, the administration of Darmocare Pre^®^ induced diarrhea, although a direct causality was not demonstrated. The total drop out number of the intervention group was eight out of 36 and was due to digestive tract symptoms such as cramps/diarrhea, whereas nearly 50% of the prebiotic group experienced mild flatulence. The majority of symptoms disappeared after a very short period of eight days, but eight participants still dropped out before symptoms vanished. No major adverse effects of the use of prebiotics are known next to the symptoms our participants suffered [40], and these effects only seem serious with an intake of more than 30 gr/day [41]. The symptoms of bloating and gaseousness are probably adaptive to chronic consumption [42], which was also made evident in our study where adverse symptoms vanished after an average of eight days. Our study has several limitations. We did not analyze several immune parameters such as IL-6 and IL-10 with which we could have researched the pro- or anti-inflammatory effects of prebiotics in the elderly, and the same holds for the absence of other immunological measurements such as subpopulations of T and B lymphocytes. The small number of participants also limited the outcomes and the development of hard conclusions. Nevertheless, our results are still promising and bigger studies using clinical hard endpoints and more biochemical and immunological parameters are warranted. Another test that we could not include was total micro biome in stool; this limitation was due to financial burden. However, the improvement of several frailty parameters through the use of prebiotics is important, and when confirmed by bigger studies, could be used not only as a cure but perhaps even as a preventive measurement.

## 4. Experimental Section

### 4.1. Study Population

Participants were recruited from individuals living in nursing homes located in Valencia province (Spain). The inclusion criteria were the ability to get up from a chair and walk 6 m, and an age of 65 years or older. The exclusion criteria were dementia, major psychiatric disease (schizophrenia, bipolar disorders, *etc.*) or blindness, acute infections, or known cancers. The present study was conducted according to the guidelines laid down in the Declaration of Helsinki, and all procedures involving human participants were approved by the Ethics Committee of the University of Valencia (Valencia, Spain) (H1424156718665). Written informed consent was obtained from all participants.

### 4.2. Intervention

The prebiotic Darmocare Pre^®^ and the placebo (maltodextrin) were physically indistinguishable. The products were always stored at room temperature. Nurses or the participants themselves dissolved one spoon of Darmocare Pre^®^ or placebo in a glass of water, and it was administered in the morning shortly after breakfast (9–10 a.m.). The intervention and placebo products were comparable in visual appearance and taste. The participants were instructed to communicate any secondary effects of gastrointestinal alteration including changes in stool consistency or defecation frequency and to maintain habitual dietary habits. The participants were interviewed about all types of potential adverse effects including the most common side effects *i.e.*, gas and cramping; in addition, they were asked specifically for changes in stool characteristics (consistency and frequency). All participants in the clinical trial were recruited from individuals living in nursing homes (as stated in the Experimental Section), in which diet was constantly supervised by a team of nutritionists/dietitians. In addition, in order to compare with the results on diet intervention, we estimated the energy intake in the study sample by assessment with a three-day food diary (one weekend and two weekdays) before the beginning of the study and twice during the intervention period. A nutritional program (using a macro procedure of a spreadsheet) including a food database for Spanish population was used.

### 4.3. Measurement of Frailty Criteria

Frailty level was measured according to the five Fried criteria [3] as previously reported by our group [2]. Briefly, the criteria were assessed as follows: (1) unintentional body weight loss (5 percent or 4.5 kg or more in the last year); (2) self-reported chronic fatigue: participants met the criteria if they answered “A few times”, “Often”, or “Most of the time” to the question “How often in the last week did you feel that everything you did was an effort?”, included in the Center for Epidemiologic Studies depression scale [43]; (3) low physical activity level was measured using the Spanish adaptation of the Minnesota leisure time physical activity questionnaire [44,45,46]; (4) according to the standards of the short physical performance battery [47], participants who walked 4.6 m in a longer time than the worst quintile of the sex and height-adjusted sample fulfilled the reduced walking speed criterion; our values were: men taller than 173 cm: ≥6 s, height < 173 cm: ≥7 s; women taller than 159 cm: ≥6 s, height < 159 cm: ≥7 s; and (5) to measure muscle weakness, grip strength (Kg) was measured three times in each hand alternately with a hydraulic dynamometer (Jaymar, J.A. Preston, Corp., Jackson, MS, USA) according to the standards of the Hispanic established populations for epidemiologic studies of the elderly [48]. Participants were considered frail if they met at least three criteria and prefrail if they met one or two. All measurements were performed by trained members of the Department of Nursing at the University of Valencia, using a questionnaire with detailed instructions.

### 4.4. Geriatric Assessment

Functional status was evaluated using the Barthel index that defines the ability to perform the basic activities of daily living [49] and measures the level of independence related to the following 10 items: feeding, bathing, dressing, grooming, defecating, urinating, toilet use, transfers (e.g., from armchair to bed), walking, and climbing stairs. The index has a score range 0–100, where 0 is total dependence and 100 corresponds to total independence. The MMSE test was used to detect cognitive impairment. It evaluates different items grouped into five sections: orientation, immediate memory, attention and calculation, delayed recall, and language and construction with a score range of 0–30, the highest scores indicating better performance [38]. Another important issue evaluated was sleep quality using the Athens Insomnia Scale (AIS) [50]. It consists of eight items related to sleep induction, awakenings during the night, final awakening, total sleep duration, sleep quality at night, well-being, functioning capacity, and sleepiness during the day. Each item of the AIS can be rated 0–3, (with 0 corresponding to “no problem at all” and 3 “very serious problem”). The scale has a score range 0–24, where 0 denotes absence of any sleep-related problem and 24 means the most severe degree of insomnia.

### 4.5. Blood Analytical Parameters

Blood samples were obtained from each subject between 7:30 a.m and 10 a.m under at least 8 h in fasting conditions. Blood was collected by collecting 10 mL blood each into two Becton Dickinson (BD) Vacutainer tubes (Becton Dickinson, Franklin Lakes, NJ, USA) containing ethylenediaminetetraacetic acid sodium salt (EDTA). After extraction, the blood samples were allowed to stand for 15 min and were centrifuged at 1500 rpm for 10 min at room temperature. Subsequently the plasma supernatants were aliquoted and stored at −20 °C until analysis. After thawing, the samples were centrifuged at 1500 rpm for 10 min at room temperature to completely remove all cells. For all other analytical determinations, residential center control blood extractions were used. Hematological parameters (white blood cells, hemoglobin, erythrocytes, and platelets) and biochemical parameters (glucose, urea, urate, cholesterol, triglycerides, creatinine, glutamic oxaloacetic transaminase (GOT), and serum glutamic pyruvic transaminase (GPT), and C-reactive protein) were measured in clinical laboratories belonging to local public health centers. Hematologic analysis included total red blood cell count (RBC) and white blood cell count (WBC) counts obtained by hemocytometer methodology (Autocrit Ultra3 Centrifuge, Becton Dickinson). Serum analytic values were determined on a laboratory chemistry analyzer (Dimension Xpand Plus Integrated Chemistry System, Siemens, Erlangen, Germany). Serum concentration of TNF-α was measured using a commercial enzyme-linked immunosorbent assay kit according to the manufacturer’s instructions (TNF-α (ab100654), Human ELISA Kit, Abcam^®^, Cambridge, UK). To minimize assay variance, all the measurements were conducted in duplicates and on the same day.

### 4.6. Statistical Analysis

Descriptive statistics, including measurements of central tendency (mean), median, and range values, were used to describe all the quantitative variables. The normal distribution of each variable was estimated with the Kolmogorov–Smirnov test. The non-parametric Mann–Whitney U test was performed to verify any possible differences between the two experimental groups. Statistical significance was set at *p* < 0.05 and statistical analysis was performed with the software SPSS 21.0 (SPSS Inc., Chicago, IL, USA).

## 5. Conclusions

The use of prebiotics such as Darmocare Pre^®^ in older individuals influences certain frailty symptoms and improves the levels of fatigue and muscle strength. Prebiotics could therefore be included in the total treatment protocol of people suffering especially from frailty, and as a preventive intervention in general. Nevertheless, it is necessary to identify those individuals who would benefit the most from the use of prebiotics through selected randomized trials.

## Figures and Tables

**Table 1:** Frailty criteria and geriatric evaluation at baseline and post-treatment with Darmocare Pre^®^ or placebo. * *p* < 0.05; ** *p* < 0.01.

Variable	Baseline		Significance	Post-Treatment		Significance
	Placebo Group (*n* = 22)	Dermocare Pre^®^ (*n* = 28)	*p* Value	Placebo Group (*n* = 22)	Dermocare Pre^®^ (*n* = 28)	*p* Value
Exhaustion (score 0–3: 0 “never”; 1 “A few times” (1–2 days per week); 2 “Often” (3–4 days per week); or 3 “Most of the time” (almost each day))	1.1 ± 1.7	1.4 ± 1.7	0.74	1.7 ± 1.2	0.8 ± 1.4 **	0.002
Slow walk (s) (time needed to walk 4.6 m)	8.6 ± 9.0	8.4 ± 6.0	0.91	8.7 ± 4.2	7.9 ± 4.5	0.48
Grip strength (right hand, kg)	11.5 ± 5.7	10.6 ± 8.2	0.61	10.2 ± 4.1	12.4 ± 3.2 *	0.04
Grip strength (left hand, kg)	10.2 ± 5.8	10.1 ± 7.6	0.92	9.1 ± 3.7	9.8 ± 3.5	0.50
Self health-perception (score 0–10, being 0 the worst and 10 the best)	7.1 ± 2.3	7.1 ± 2.1	0.96	6.8 ± 2.4	6.8 ± 2.0	0.96
Body mass index	26.1 ± 4.1	25.8 ± 4.2	0.97	26.0 ± 3.8	25.9 ± 4.1	0.96
Athens insomnia scale	3.4 ± 3.0	4.1 ± 4.7	0.77	4.5 ± 5.3	4.0 ± 4.3	0.68
Barthel index	76.2 ± 13.0	74.6 ± 17.7	0.69	78.3 ± 13.9	77.1 ± 29.9	0.87
Mini–Mental state examination	26.1 ± 2.2	26.5 ± 3.1	0.89	25.9 ± 2.1	26.4 ± 2.2	0.85

**Table 2:** Blood analysis and haemogram after treatment with Darmocare Pre^®^ or the placebo. GOT: glutamic oxaloacetic transaminase; GPT: glutamic-pyruvate transaminase; HDL: high-density lipoproteins; LDL: low-density lipoproteins; TNF: tumor necrosis factor α.

Variable	Placebo Group (*n* = 22)	Darmocare Pre^®^ (*n* = 28)	*p* Value
Leukocytes (×10^3^/µL)	7.6 ± 0.5	7.7 ± 0.8	0.92
Neutrophils (×10^3^/µL)	4.5 ± 0.2	4.6 ± 0.3	0.79
Lymphocytes (×10^3^/µL)	2.2 ± 0.2	2.3 ± 0.1	0.64
Monocytes (×10^3^/µL)	0.61 ± 0.04	0.55 ± 0.03	0.23
Eosinophils (×10^3^/µL)	0.22 ± 0.04	0.22 ± 0.05	0.88
Basophils (×10^3^/µL)	0.03 ± 0.01	0.03 ± 0.01	1.00
Platelets (×10^3^/µL)	220 ± 28	225 ± 36	0.92
Erythrocytes (×10^6^/µL)	5.0 ± 0.6	4.8 ± 0.7	0.83
Haemoglobin (g/dL)	12.8 ± 1.3	12.9 ± 1.1	0.95
Glucose (mg/dL)	95 ± 11	92 ± 10	0.84
Urea (mg/dL)	44 ± 5	40 ± 3	0.48
GOT (U/L)	28 ± 3	27 ± 4	0.85
GPT (U/L)	25 ± 2	24 ± 4	0.84
HDL cholesterol (mg/dL)	46 ± 6	44 ± 7	0.83
LDL cholesterol (mg/dL)	120 ± 9	126 ± 11	0.69
Triglycerides (mg/dL)	131 ± 21	122 ± 16	0.73
Total proteins (g/dL)	7.5 ± 0.4	7.4 ± 0.5	0.88
Creatinine (mg/dL)	0.71 ± 0.10	0.74 ± 0.15	0.88
Calcium (mg/dL)	8.6 ± 1.0	8.9 ± 0.8	0.81
Sodium (mEq/L)	140 ± 3	141 ± 3	0.82
Potassium (mEq/L)	4.7 ± 0.8	4.8 ± 0.5	0.91
C-reactive protein (mg/L)	4.8 ± 1.5	4.9 ± 1.8	0.97
TNF-α (pg/mL)	1.8 ± 0.2	2.0 ± 0.3	0.60
